# Cost-effectiveness of time-lapse monitoring with or without the use of embryo selection software compared to routine incubation and selection

**DOI:** 10.1093/hropen/hoag034

**Published:** 2026-04-22

**Authors:** Dorit C Kieslinger, Carlijn G Vergouw, Liliana Ramos, Brigitte Arends, Max H J M Curfs, Els Slappendel, E Hanna Kostelijk, Math H E C Pieters, Dimitri Consten, Marieke O Verhoeven, Dagmar E Besselink, Frank Broekmans, Ben J Cohlen, Jesper M J Smeenk, Sebastiaan Mastenbroek, Corry H de Koning, Yvonne M van Kasteren, Etelka Moll, Jeroen van Disseldorp, Egbert A Brinkhuis, Esther A M Kuijper, W Marchien van Baal, Hans G I van Weering, Paul J Q van der Linden, Marie H Gerards, Patrick M Bossuyt, Cornelis B Lambalk, Madelon van Wely

**Affiliations:** IVF Center, Amsterdam UMC—Location VUmc, Amsterdam, The Netherlands; IVF Center, Amsterdam UMC—Location VUmc, Amsterdam, The Netherlands; IVF Center, Radboudumc, Nijmegen, The Netherlands; IVF Center, UMC Utrecht, Utrecht, The Netherlands; Isala Fertility Center, Isala, Zwolle, The Netherlands; Center for Fertility, Nij Geertgen, Elsendorp, The Netherlands; IVF Center, Amsterdam UMC—Location VUmc, Amsterdam, The Netherlands; IVF Center, UMC Utrecht, Utrecht, The Netherlands; IVF Center, ETZ, Tilburg, The Netherlands; IVF Center, Amsterdam UMC—Location VUmc, Amsterdam, The Netherlands; IVF Center, Radboudumc, Nijmegen, The Netherlands; IVF Center, UMC Utrecht, Utrecht, The Netherlands; Isala Fertility Center, Isala, Zwolle, The Netherlands; IVF Center, ETZ, Tilburg, The Netherlands; Center for Reproductive Medicine, Amsterdam UMC—Location AMC, Amsterdam, The Netherlands; Center for Fertility, Tergooi MC, Blaricum, The Netherlands; Center for Fertility, NWZ, Alkmaar, The Netherlands; Center for Fertility, OLVG, Amsterdam, The Netherlands; Center for Fertility, St. Antonius, Nieuwegein, The Netherlands; Center for Fertility, Meander MC, Amersfoort, The Netherlands; Center for Fertility, Spaarne Gasthuis, Haarlem, The Netherlands; Center for Fertility, Flevo Hospital, Almere, The Netherlands; Center for Fertility, RKZ, Beverwijk, The Netherlands; Department of Obstetrics, Gynaecology and Reproductive Medicine, Deventer Ziekenhuis, Deventer, The Netherlands; Center for Fertility, Diakonessenhuis, Utrecht, The Netherlands; Epidemiology & Data Science, Amsterdam UMC, Amsterdam, The Netherlands; IVF Center, Amsterdam UMC—Location VUmc, Amsterdam, The Netherlands; Center for Reproductive Medicine, Amsterdam UMC—Location AMC, Amsterdam, The Netherlands

**Keywords:** time-lapse monitoring, cost-effectiveness, IVF, embryo culture, machine learning, costs

## Abstract

**STUDY QUESTION:**

Is time-lapse monitoring (TLM) with or without a machine learning selection algorithm for embryo selection cost-effective when compared with conventional culture and selection?

**SUMMARY ANSWER:**

Neither TLM with or without the use of machine learning for embryo selection is likely to be cost-effective when compared with the conventional approach. TLM adds cost to IVF treatments without improving clinical results.

**WHAT IS KNOWN ALREADY:**

Recent randomized controlled trials (RCTs) provide evidence that TLM is unlikely to increase pregnancy or live birth rates (LBRs) relative to conventional embryo culture and selection. To date no cost-effectiveness analysis of TLM has been published.

**STUDY DESIGN, SIZE, DURATION:**

A cost-effectiveness analysis (CEA) from a healthcare perspective was performed based on data from our multicentre RCT on TLM, which was conducted from 2017 to 2020. Women scheduled for Day 3 single embryo transfer during their first, second, or third oocyte retrieval were included. The trial included three strategies: (i) TLE: embryo selection based on a machine learning algorithm and uninterrupted culture; (ii) TLR: routine morphological embryo selection and uninterrupted culture; and (iii) CON (Control): routine morphological embryo selection and interrupted culture.

**PARTICIPANTS/MATERIALS, SETTING, METHODS:**

In total, 1731 couples were included. The difference in mean costs between treatment groups was calculated with the following cost items: laboratory procedures, laboratory facilities (incubator, disposables, service), embryo transfers, pregnancies, miscarriages, and deliveries. We did not consider costs of stimulation, oocyte retrieval, and IVF/ICSI procedures as these were identical in each group. The effectiveness measure for the CEA was the cumulative LBR. Incremental cost-effectiveness ratios were calculated for direct comparisons between strategies.

**MAIN RESULTS AND THE ROLE OF CHANCE:**

The 12-month cumulative LBR rate was similar in the three treatment groups: TLE 48.7%, TLR 48.4%, CON 48.2%. The mean difference in costs was €237 (95% CI: −35 to 508) between TLE and CON, and €55 (95% CI: −203 to 313) between TLR and CON. The incremental cost-effectiveness ratio between TLE and CON for one additional live born was €45 950; for TLR versus CON, it was €26 905. The probability that TLE or TLR are cost-effective was low for all willingness-to-pay level, with several scenarios resulting in comparable findings.

**LIMITATIONS, REASONS FOR CAUTION:**

This study reports the cost-effectiveness for one time-lapse incubator; however, more systems are currently available.

**WIDER IMPLICATIONS OF THE FINDINGS:**

While our cost-effectiveness analysis provides evidence that TLM is unlikely to be cost-effective, its use is not inferior to conventional methods in terms of clinical results and can offer certain advantages for IVF laboratories.

**STUDY FUNDING/COMPETING INTEREST(S):**

The authors received a grant from the Netherlands Organization for Health Research and Development (ZonMw) for the execution of the SelecTIMO study (Health Care Efficiency Research programme grant 843001602). Merck (Germany and the Netherlands) supplied the six time-lapse incubators, funded the laboratory adjustments, and provided technical support and training to laboratory personnel before and during the study. The following declarations of competing interests are outside the submitted work: J.M.J.S. reports research grants from Ferring BV and Merck BV, speakers’ fees from Merck BV and travel support for attending meetings from Merck BV and Goodlife BV. C.B.L. reports speaker’s honoraria from Merck and Organon (The Netherlands), travel support from Merck and Organon, and was Editor-in-Chief of *Human Reproduction*. M.v.W. is Editor-in-Chief of *Human Reproduction Update*. The remaining authors have no conflicts of interest to declare.

**TRIAL REGISTRATION NUMBER:**

NTR5423: ICTRP Search Portal (who.int).

WHAT DOES THIS MEAN FOR PATIENTS? During IVF treatment, embryos are kept in incubators that create the right conditions for them to grow. In the standard method, embryologists take the embryos out of the incubator once a day to check them under a microscope. Based on these checks, the embryo that is most likely to lead to a pregnancy is placed in the uterus.

Today, many IVF laboratories use a different type of incubator called a time-lapse incubator. This incubator has a camera inside. It takes pictures of the embryos as they grow, so embryologists can follow their development without taking them out of the incubator.

Our study looked at the added benefits and costs of time-lapse incubators compared with the standard method. We found that the chance of having a baby within 12 months was similar for time-lapse incubators and standard incubators. However, time-lapse incubators are more expensive. The use of time-lapse monitoring is unlikely to be cost-effective, meaning that the higher costs are not balanced by better outcomes.

For patients, this means that time-lapse technology does not increase the chance of having a baby, but it does increase the cost of IVF treatment. The standard method of growing and selecting embryos therefore remains the most cost-effective option.

## Introduction

Time-lapse incubators have been introduced to IVF laboratories worldwide before randomized controlled trials (RCT) on clinical benefits were performed (Perrotta and Geampana, 2020). One of the advantages of time-lapse monitoring (TLM) is that it facilitates continuous monitoring of embryo development without the need to remove the embryo dishes from the stable culture environment of the incubator. Moreover, machine learning, deep learning, and artificial intelligence can be used to evaluate the data and select embryos for transfer and cryopreservation. Several large RCTs and a Cochrane review concluded that the suggested advantages of TLM do not appear to improve clinical results, with similar pregnancy and live birth rates (LBRs) being reported for TLM and conventional culture and embryo selection methods (Armstrong *et al.*, 2019; Ahlstrom *et al.*, 2022; Kieslinger *et al.*, 2023; Bhide *et al.*, 2024; Illingworth *et al.*, 2024). Furthermore, TLM does not lead to a higher cumulative LBR within 1 year or reduce the time to pregnancy, compared to routine embryo culture and selection (Kieslinger *et al.*, 2023).

In addition to evaluating their clinical effectiveness, technological innovations in IVF should be critically assessed in terms of safety, workflow efficiency, and costs (Kieslinger *et al.*, 2024). TLM has been shown to be safe in terms of obstetric and neonatal risks (Kieslinger *et al.*, 2026). Steps to improving workflow efficiency in an IVF laboratory may include reducing laboratory procedure times (while maintaining high quality), optimizing laboratory protocols, streamlining processes, advancing automation, reducing administrative workload, and optimizing laboratory resource use. A recent study provides evidence that TLM can lead to a 10-fold reduction in embryo evaluation time (Illingworth *et al.*, 2024), which could potentially decrease costs of IVF treatments and contribute to increasing workflow efficiency. However, a balanced evaluation of an innovation in the field of IVF should include a thorough cost-effectiveness analysis (ESHRE Capri Workshop Group, 2015; ESHRE Add-Ons Working Group *et al.*, 2023; Feng *et al.*, 2024; Kieslinger *et al.*, 2024). We hypothesized that the application of TLM would result in an increased cumulative live birth pregnancy rate, thereby balancing the additional costs associated with its implementation and use. The aim of this study was to evaluate the cost-effectiveness of TLM from a healthcare perspective based on data from our multicentre RCT on TLM.

## Materials and methods

### Study design

An economic evaluation with a cost-effectiveness analysis was conducted alongside our multicentre, three-armed, double-blind RCT on TLM (Kieslinger *et al.*, 2023). Couples were included at 15 fertility clinics in the Netherlands. Participating couples provided written informed consent. The laboratory procedures were performed in five IVF laboratories. Randomization was done centrally using Castor and stratified for laboratory and oocyte retrieval cycle number. The study was registered at Trial Search on 8 September 2015 (NTR5423: ICTRP Search Portal (who.int)). The trial protocol was first approved on 22 December 2016 by the Central Committee on Research involving Human Subjects (The Hague, The Netherlands) and by the board of directors of each participating clinic. The SelecTIMO study compared the following three groups: (i) TLE (Time-Lapse Eeva): embryo selection based on the Eeva^®^ Test (a Day three TLM algorithm, used adjunctively with morphology) and uninterrupted culture; (ii) TLR (time-lapse routine): routine morphological embryo selection and uninterrupted culture; and (iii) CON (Control): routine morphological embryo selection and interrupted culture. In the TLE and TLR group, zygotes were transferred to Geri+ culture dishes after fertilization check on Day 1 and cultured uninterruptedly until embryo transfer on Day 3. In the CON group, standard dishes were used for embryo culture and dishes were removed for morphological assessment two additional times between Day 1 and embryo transfer on Day 3, thereby providing interrupted embryo culture. All embryos were cultured in the Geri+ time-lapse incubator (Genea Biomedx, Sydney, NSW, Australia) until the moment of cryopreservation. Outcomes of fresh and frozen embryo transfers associated with the initial oocyte retrieval cycle within 1 year, as well as natural conceptions, were recorded. The three-armed design enabled a distinction between the effect of the time-lapse-based selection method and the contribution of the uninterrupted culture conditions, which are an element of this technique. Additional details concerning participants, randomization, and IVF protocols can be found in our previously published article (Kieslinger *et al.*, 2023).

### Unit costs

Differences in mean costs between the strategies were calculated from the health care perspective with the following cost items: laboratory procedures, laboratory facilities (incubator, disposables, service), embryo transfers, pregnancies, miscarriages, and deliveries within 12 months after randomization. We did not consider the costs of ovarian stimulation, oocyte retrieval, and IVF/ICSI procedures as these were identical in each group.

We recorded the time needed for laboratory procedures in the three groups for the following elements: preparation of standard dishes and Geri dishes, registration of patient data and alignment of dishes in Geri incubator, placement of 2PN zygotes in culture dishes, embryo assessments from Day 1 until Day 3 on screen or under standard microscope, and review and manual update of time-lapse videos. Time measurements for the laboratory procedures were performed at the end of the study period in one of the five IVF laboratories. The following IVF/ICSI procedures were identical for the three strategies and were not documented: semen preparation, oocyte retrieval, insemination using IVF or ICSI, fertilization score, embryo cryopreservation, and embryo thawing.

Costs for laboratory personnel were based on the collective employment agreement of the university medical centres in the Netherlands for the year 2020 (IVF technician in salary scale 8, incremental pay rise 10). We multiplied the time needed for laboratory procedures, depending on the number of fertilized embryos per couple, with the hourly rate for the laboratory personnel.

Prices for laboratory facilities were derived from quotations for incubators, disposables, service-level agreements, and licenses (if applicable). Costs were calculated for the Geri+ time-lapse incubator with application of the Eeva Test for embryo selection in the TLE group and without application of the Eeva test in the TLR group. For the SelecTIMO study, all embryos were cultured in Geri+ incubators, but we selected two standard incubators to represent conventional incubation in the control group. Costs for this standard incubator were calculated for the Miri Multi-Room (Esco, MRI-6A10) and Nuaire (NU57410E) incubators, with similar costs for both incubators. The following costs were included in our calculations: initial purchase costs for incubators, annual maintenance costs, culture dishes and in case of the Geri+ time-lapse incubators, costs for server and software per patient (Eeva Test embryo selection prediction). The costs for monitoring of gas and temperature conditions were not included. All costs were based on quotations from manufacturers issued in 2020/2021.

Costs for the number of embryo transfers, pregnancies, miscarriages, and deliveries (singleton and multiple gestation births) within the follow-up period of 1 year were based on a study by Lukassen *et al.* (2004) and indexed to 2020 price levels.

Fertility treatment (embryo transfers, insemination method) and pregnancy loss costs (miscarriage and ectopic pregnancy) were based on estimates from the Dutch Consortium for Research in Women’s Health, where an expert panel of gynaecologists, economists, and a methodologist derived per-unit medical costs using data from two university hospitals and one general hospital.

### Statistical analyses

The cumulative LBR within a 1-year time-horizon was selected as primary effectiveness measure according to the following definition: a live birth following an ongoing pregnancy conceived within 12 months after randomization, including pregnancies achieved via fresh embryo transfer or frozen embryo transfer derived from the initial oocyte retrieval cycle, or natural conception. Costs were analysed using generalized linear models with a gamma distribution and identity link to model mean costs over the 1-year time horizon, adjusted for the stratification factors (laboratory and cycle number).

We compared TLE and TLR with the control (CON). We first calculated the difference in mean total costs and subsequently the incremental cost-effectiveness ratio (ICER) as the difference in mean total costs divided by the difference in live birth probability, adjusted for the stratification variables. We obtained 95% confidence intervals for costs outcomes and effects by applying bootstrapping with 5000 resamples. The ICER results were plotted in a cost-effectiveness plane, which illustrates the four possible cost-effectiveness outcomes.

Using the same bootstrap samples, we constructed a cost-effectiveness acceptability curve (CEAC) across willingness-to-pay thresholds from €0 to €100 000 (expressed per steps of €10 000). The CEAC shows the probability that TLE or TLR is cost-effective at each threshold, reflecting uncertainty even when estimates fall across multiple quadrants.

Several scenario analyses and sensitivity analyses were performed. A first scenario evaluated cost-effectiveness for a shorter incubator depreciation period of 3 years, instead of 10 years. In a second scenario, cost-effectiveness was analysed for an optimized usage of TLM. We extrapolated the minimum time needed in each treatment group when embryos are cultured uninterruptedly until Day 5. In this scenario, embryos would only be assessed on Day 1 and Day 5, which would result in less time needed per embryo in the TLE and TLR groups than in the CON group. In our third scenario, we used a 10-fold reduction in evaluation time in the TLE group compared to CON group for culture until Day 5.

We performed a sensitivity analysis by including all costs until an ongoing pregnancy, excluding all perinatal and delivery costs. As age may impact both effectiveness and cost, we also evaluated the impact of age on cost-effectiveness by including age and age*treatment interaction in the model.

The databases were prepared in SPSS and the analyses were done in STATA 19 (Stata Statistical Software: Release 19. StataCorp LLC, College Station, TX, USA).

## Results

### Effectiveness outcomes

Between 15 June 2017 and 31 March 2020, a total of 1731 couples were randomly allocated to one of the three study groups. Cumulative pregnancy and live birth results and absolute differences of TLE and TLR compared to CON are depicted in Table 1. The cumulative LBR at 12 months was 48.7% (281 of 577) in the TLE group, 48.4% (280 of 579) in the TLR group, and 48.2% (277 of 575) in the control group, with no significant differences among the three treatment groups (*P* = 0.98).

**Table 1.** Pregnancy outcomes after 1 year in all three groups.

Cumulative results	TLE group	TLR group	CON group	Absolute difference TLE versus CON	Absolute difference TLR versus CON
Cumulative positive hCG rate	357/577 (61.9*%*)	355/579 (61.3*%*)	357/575 (62.1*%*)	−0.22% (−6.56 to 6.13)	−0.77% (−7.11 to 5.57)
Cumulative clinical pregnancy rate	328/577 (56.9*%*)	336/579 (58.0*%*)	328/575 (57.0*%*)	−0.20% (−6.66 to 6.26)	0.99% (−5.47 to 7.44)
Cumulative ongoing pregnancy rate	293/577 (50.8*%*)	295/579 (51.0*%*)	284/575 (49.4*%*)	1.39% (−5.14 to 7.92)	1.56% (−4.96 to 8.08)
Cumulative live birth rate	281/577 (48.7*%*)	280/579 (48.4*%*)	277/575 (48.2*%*)	0.53% (−6.00 to 7.05)	0.19% (−6.33 to 6.71)
Cumulative miscarriage rate	92/577 (15.9*%*)	94/579 (16.2*%*)	100/575 (17.4*%*)	−1.45% (−6.29 to 3.40)	−1.16% (−6.00 to 3.69)

TLE, time-lapse early embryo viability assessment; TLR, time-lapse routine; CON, control.

### Economic evaluation

The cost items included in our cost-effectiveness analysis are shown in Table 2. More time was needed in both time-lapse monitoring groups (TLE and TLR) compared to the conventional method (CON). In addition, the acquisition, maintenance, and disposables costs of a time-lapse incubator were higher than those of a standard incubator.

**Table 2.** Cost items and resource use per woman.

Cost item	Unit cost (Euro)	Reference	TLE	TLR	CON
***Time usage for laboratory procedures per embryo***					
**Total time in seconds Days 1–3** (model)	0.02 per second	Based on Dutch price levels in 2020 (CAO UMC: 8–10)	250.5 (44.5)	178.8 (12.1)	152.9 (43.3)
**Total time in seconds** (scenario 2, optimized culture till Day 5)			178.8	107.1	233.6
**Total time in seconds** (scenario 3, culture till Day 5, 10-fold reduction based on results of Illingworth *et al.*, 2024)			23.6	23.6	233.6
***Laboratory facilities (for 5 Geri or standard incubators)*** *					
**Incubator, disposables, service** (10 years: model)			162	59	9
**Incubator, disposables, service** (3 years: scenario 1)			227	123	21
***IVF/ICSI***					
**Embryo transfer**	386.1	Dutch Consortium	1.92 (1.51) Median 1 (range 0–9)	1.94 (1.54) Median 1 (range 0–9)	1.94 (1.50) Median 1 (range 0–9)
**Pregnancy and delivery**					
Singleton	3424.5	Lukassen *et al.* (2004)	273	276	274
Twin	18 096.9	Lukassen *et al.* (2004)	6	4	3
Miscarriage	100.3	Lukassen *et al.* (2004)	92	94	100

* Costs per woman for time-lapse and standard incubators were calculated based on the following prizes given by manufacturers in 2020/2021.

**Time-lapse system (Geri):** Initial purchase cost per Geri incubator, €80 000; Eeva server, €3500; annual Geri maintenance, €6000; Eeva prediction per patient (TLE group), €100; Geri culture dish per patient, €15.

**Standard incubation:** Miri multi incubator (MRI-6A10) including inserts, €17 720; annual maintenance, €700; Nuaire incubator (NU57410E) including segmented inner doors, €11 024; annual maintenance, €1700; standard Nunc IVF dish (60 mm), €0.35. Average purchase price of both standard incubator types used to calculate per-patient costs.

TLE, time-lapse early embryo viability assessment; TLR, time-lapse routine; CON, control. Data are mean (SD).

### Costs

Table 3 reports mean costs, cost differences, and ICERs between the groups. In the base model with an incubator depreciation period of 10 years, the mean difference in costs until a live birth was €237 (95% CI: −35 to 508) between TLE and CON, and €55 (95% CI: −203 to 313) between TLR and CON. When considering costs until an ongoing pregnancy, mean differences were €148 (95% CI: 81–214) between TLE and CON, and €50 (95% CI: −18 to 118) between TLR and CON. There was no significant effect of the stratification factors laboratory and cycle number on the mean cost differences; costs per stratification factor for the main models are reported in Supplementary Table S1.

**Table 3.** Scenario and sensitivity analyses.

					TLE vs CON		TLR vs CON	
Scenario	Description	Mean cost TLE	Mean cost TLR	Mean cost CON	Bootstrapped cost difference	ICER	Bootstrapped cost difference	ICER
Base model	Cumulative live birth (Incubator depreciation period of 10 years)	€2797(2594 to 3001)	€2615(2429 to 2801)	€2560(2387 to 2733)	€237(−35 to 508)	€45 950(−115 149 to 145 639)	€55(−203 to 313)	€26 905(−43 898 to 61 781)
Scenario 1	Cumulative live birth rate (Incubator depreciation period of 3 years)	€2850(2652 to 3049)	€2668(2481 to 2855)	€2560(2387 to 2733)	€289(−9 to 586)	€56 333(−113 472 to 154 989)	€108(−160 to 375)	€54 188(−64 054 to 84 591)
Scenario 2	Cumulative live birth rate (optimized culture till Day 5 TLE/TLR) 10 years	€2780(2383 to 2737)	€2599(2420 to 2780)	€2560(2387 to 2733)	€213(−50 to 476)	€42 870(−118 027 to 141 130)	€39(−213 to 291)	€20 133(−37 423 to 55 974)
Scenario 3	Cumulative live birth rate (10-fold reduction in evaluation time TLE/TLR)	€2768(2565 to 2971)	€2590(2407 to 2775)	€2560(2387 to 2733)	€208(−78 to 484)	€39 825(−105 403 to 135 403)	€30(−225 to 284)	€15 008(−32 777 to 48 691)
Sensitivity analysis until OP	Ongoing pregnancy (Incubator depreciation period of 10 years)	€938(891 to 988)	€841(793 to 889)	€791(743 to 839)	€148(81 to 214)	€10 459(−65 638 to 91 149)	€50(−18 to 118)	€3152(−25 701 to 29 655)
Sensitivity analysis accounting for age	Base model including age and age*treatment	€2787(2585 to 2989)	€2617(2342 to 2892)	€2558(2393 to 2723)	€229(−24 to 483)	€428 065(−835 690 to 1 691 820)	€61(−173 to 295)	€−13 127(−701 427 to 675 172)

TLE, time-lapse early embryo viability assessment; TLR, time-lapse routine; CON, control; ICER, incremental cost-effectiveness ratio; OP, ongoing pregnancy.

### Cost effectiveness

**Figure 1.**
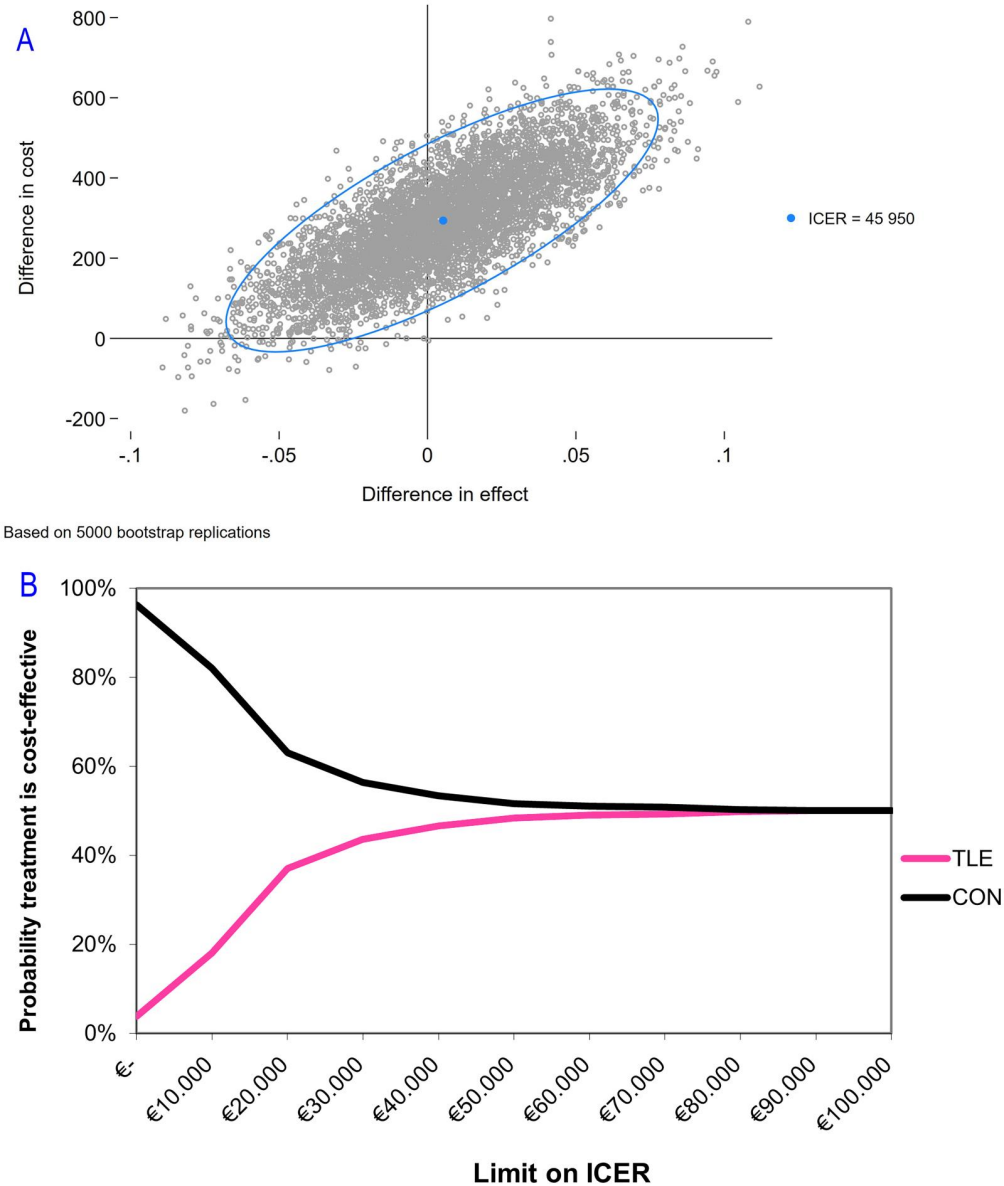
**Cost-effectiveness graphs for the comparison of TLE versus CON**. (**A**) Cost-effectiveness plane for the application of time-lapse monitoring with the use of a machine learning algorithm for embryo selection (TLE) versus conventional embryo culture and selection (CON). Each point in the cost-effectiveness plane represents the uncertainty of the additional costs and effect (measured as live birth within 1 year) of time-lapse monitoring versus conventional embryo culture after non-parametric bootstrap resampling (5000 samples). **(B**) Cost-effectiveness acceptability curve (CEAC) for the application of time-lapse monitoring with the use of a machine learning algorithm for embryo selection (TLE) versus conventional embryo culture and selection (CON). The CEAC provides more information on the uncertainty surrounding the cost-effectiveness. Costs are expressed in Euros. ICER, incremental cost-effectiveness ratio.

Figure 1A shows the results for the bootstrap samples in the cost‐effectiveness plane. A comparison between the TLE and CON strategy shows that almost all estimates are located around the zero difference in effectiveness with higher costs for TLE compared to CON. Overall, 56.8% of the bootstrap estimates are located in the northeast quadrant, where TLE is more effective and more expensive while 0% are in the southeast quadrant (TLE is more effective and less expensive, ‘dominant’), 42.2% in the northwest (TLE is less effective and more expensive), and 1.0% in the southwest (TLE is less effective and less expensive). The estimated ICER was €45 950; this reflects the extra costs needed to achieve one additional live birth in couples treated with TLE, compared to the CON strategy. We calculated the 95% CI of the ICER estimate, however, because of crossing quadrants, these are difficult to interpret.

The CEAC in Fig. 1B provides more information on the uncertainty surrounding the cost-effectiveness. We found that at a willingness to pay of €20 000 per additional live birth, TLE-based management was cost-effective in 37.0% of bootstrap samples compared to CON. This increased to 48.4% for a willingness-to-pay threshold of €50 000 and 50.0% for a willingness-to-pay threshold of €100 000.

**Figure 2.**
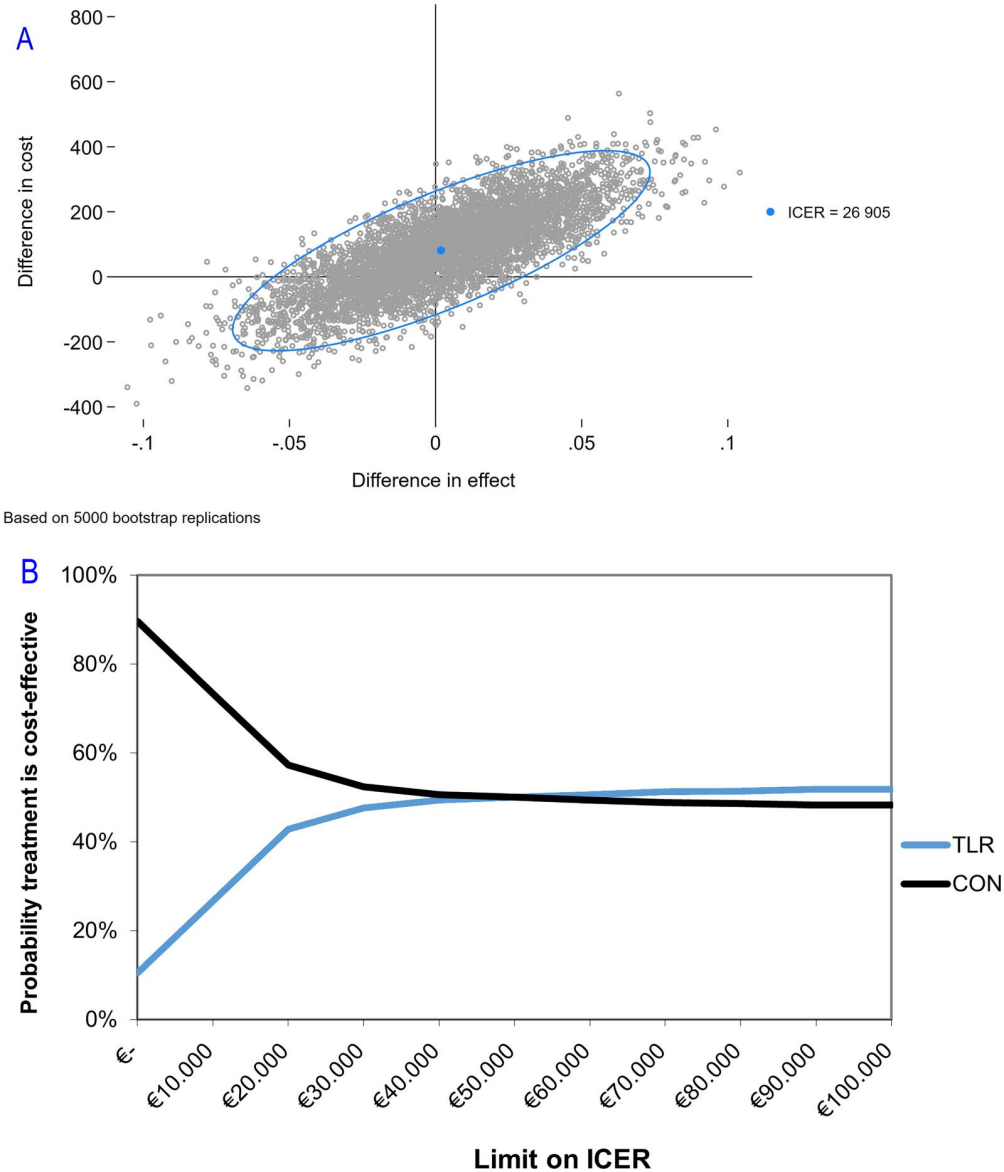
**Cost-effectiveness graphs for the comparison of TLR versus CON**. (**A**) Cost-effectiveness plane for the application of time-lapse monitoring without the use of a machine learning algorithm for embryo selection (TLR) versus conventional embryo culture and selection (CON). Each point in the cost-effectiveness plane represents the uncertainty of the additional costs and effect (measured as live birth within 1 year) of time-lapse monitoring versus conventional embryo culture after non-parametric bootstrap resampling (5000 samples). (**B**) Cost-effectiveness acceptability curve for the application of time-lapse monitoring without the use of a machine learning algorithm for embryo selection (TLR) versus conventional embryo culture and selection (CON). The CEAC provides more information on the uncertainty surrounding the cost-effectiveness. Costs are expressed in Euros. ICER, incremental cost-effectiveness ratio.

For the comparison between TLR and the CON strategy (Fig. 2A), most dots scatter around a zero difference in effectiveness and costs. Of all samples, 52.4% are located in the northeast quadrant (TLR is more effective than control and also more expensive), 4.1% in the southeast (TLR is more effective and less expensive, ‘dominant’), 23.2% in the northwest (TLR is less effective and more expensive), and 20.3% in the southwest quadrant (TLR is less effective and less expensive). The estimated ICER was €26 905, which specifies the extra costs necessary to achieve one additional live birth with TLR. We calculated 95% CI of the ICER estimate, however, because of crossing all quadrants, these are difficult to interpret.

The CEAC in Fig. 2B provides more information on the uncertainty surrounding the cost-effectiveness. We found that at a willingness-to-pay of €20 000 per additional live birth, TLR-based management was cost-effective in 42.8% of bootstrap samples, compared to CON, which increased to 50.0% for a willingness-to-pay threshold of €50 000 and 51.8% for a willingness-to-pay threshold of €100 000.

### Scenario and sensitivity analyses

The results of the scenario analyses are shown in Table 3. The ICER graphs and CEACs can be found in Supplementary Figs S1, S2, S3, S4, S5, S6, S7, and S8. The three scenario analyses resulted in similar findings compared to the main model. A shorter incubator depreciation period of 3 years instead of 10 years resulted in a larger ICER for both TLE and TLR versus CON (Scenario 1, Supplementary Figs S1 and S5). Optimized usage of TLM and culture until Day 5 (Scenario 2, Supplementary Figs S2 and S6) and a 10-fold reduction in evaluation time in the TLE group/TLR group (Scenario 3, Supplementary Figs S3 and S7) resulted in smaller ICERs for both TLE and TLR versus CON. In all cases, large uncertainty around the estimates remained and the CEACs showed largely similar results as the main model.

In the sensitivity analysis including costs until an ongoing pregnancy while excluding costs associated with childbirth, results were similar to the primary analyses (Supplementary Figs S4 and S8). ICER estimates were much lower: €10 459 for TLE versus CON and €3152 for TLR versus CON with large uncertainty around the estimates.

The sensitivity analysis in which we accounted for age in the model did not affect cost differences but resulted in a smaller absolute difference in cumulative LBR of 0.054% (95% CI: −5.65% to 5.76%) for TLE versus CON. Consequently, the ICER estimate was about 10 times as large (€428 065). For TLR versus CON, the age-adjusted absolute difference in cumulative LBR was −0.39% (95% CI: −6.03% to 5.25%) resulting in a negative ICER with the estimate in the north-west quadrant (€-3127). The willingness-to-pay remained far beneath 50% for any threshold for both comparisons.

## Discussion

We performed an economic evaluation with cost-effectiveness analysis based on our RCT on TLM to evaluate whether the use of a time-lapse incubator for embryo culture and selection is justifiable from a health care perspective. We found no clear difference in effectiveness, with higher costs for TLM at both strategies, meaning that the use of TLM is unlikely to be cost-effective compared to the conventional strategy. ICERs of €45 950 and €26 905 are reported for one additional live birth in the TLE and TLR group, respectively. The probability that TLE or TLR are cost-effective remained low at all considered willingness-to-pay levels.

It should be noted that there is no consensus in the literature regarding the willingness-to-pay threshold for a treatment in assisted reproduction. The threshold may also vary between countries (Keller and Chambers, 2022; van Hoogenhuijze *et al.*, 2022). The authors of a simplified economic evaluation to evaluate infertility treatments suggest a reference threshold for standard IVF treatment resulting in one live birth, which can be used for a more objective assessment of the cost-effectiveness of new techniques (Feng *et al.*, 2024). Applying this threshold to our data reveals that the costs of using TLM for one additional live birth falls above the proposed willingness-to-pay threshold (of €27 000) for the TLE group and just below this willingness-to-pay threshold for the TLR group. However, the CEAC suggested that at €27 000, the probability that TLE was cost-effective was only 46%, thus supporting that neither TLM method is cost-effective compared with the control. The simplified model does also not account for the extreme insecurities around the estimates due to the crossing of multiple quadrants.

We performed several scenario analyses. A shorter incubator depreciation period of 3 years resulted in comparable outcomes (Scenario 1). Furthermore, we extrapolated observation times in all three groups for uninterrupted culture until Day 5 and optimized usage of TLM since this policy is applied in most laboratories nowadays (Scenario 2). Findings from the cost-effectiveness analysis remained largely the same. Total time spent for embryo assessment was highest with TLE because manual updates and correction of annotations had to be performed by laboratory personnel. Illingworth *et al.* (2024) report a 10-fold reduction in observation time in their trial when using a deep learning algorithm, iDAscore 1.0, compared to standard methods. Modern artificial intelligence algorithms do not require substantial manual correction compared to the machine learning algorithm used in our study, which explains the difference in observation times. Illingworth *et al.* report a mean evaluation time of 21.3 s per embryo when using iDAscore 1.0. In Scenario 3, we chose a 10-fold reduction in observation time in the TLE group compared to the CON group. This shorter evaluation time did not substantially change the outcomes of the cost-effectiveness analysis. This finding may seem counterintuitive, but we think it can be explained by the relatively small proportion of costs associated with embryo evaluation and selection within the overall expenses of an IVF treatment cycle. In practice, time spent by an IVF technician on embryo evaluation per day is relatively small compared to the substantial time required for other manual laboratory procedures and administrative tasks (Veiga *et al.*, 2022). In our laboratory, which performs an average of 1300 oocyte retrieval cycles per year, embryo evaluation typically requires 2-4 hours per day, which translates into a potential reduction of half of a full-time technician per year if fully automated embryo evaluation were to be implemented.

The costs for acquisition and ongoing maintenance of multiple time-lapse incubators are substantial. This illustrates why a shorter evaluation time, on its own, cannot lead to cost-effectiveness without either a considerable decrease in costs for TLM incubators, disposables, and maintenance or a higher clinical effectiveness; the use of TLM would have to decrease the number of IVF cycles needed to achieve a pregnancy, but our data as well as several other RCTs provide no evidence for such an effect (Ahlstrom *et al.*, 2022; Kieslinger *et al.*, 2023; Bhide *et al.*, 2024; Illingworth *et al.*, 2024).

It is relevant to take female age into account in economic evaluations, in view of its likely impact on effectiveness and potential impact on costs. Accounting for age resulted in a smaller difference in effectiveness, leading to substantially larger ICER for TLE versus CON; for TLR versus CON the ICER became negative, indicating that on average, TLR was associated with higher costs and lower effectiveness.

A recent systematic review summarized economic evaluations of assisted reproductive technologies (Si *et al.*, 2024). The number of studies in this area remains very limited. Ours is the first reported economic analysis of TLM. While our study provides evidence that TLM is unlikely to be cost-effective, clinical outcomes are not inferior compared to conventional methods and TLM can offer certain advantages. The challenge in evaluating technological innovations in IVF laboratories is that their advantages may not be fully quantifiable using traditional outcome measures from RCTs alone. Modern time-lapse incubators offer several benefits over conventional methods: the continuous monitoring of embryos with minimal disturbances reduces handling of embryos, and thereby risks, and helps to maintain a stable culture environment. This may lead to epigenetic benefits, potentially manifesting later in life. Embryologists can use the detailed information on embryo development from the TLM videos and morphokinetic parameters for their decisions on embryo transfer, cryopreservation, and the timing of procedures, such as embryo biopsy. In addition, IVF laboratories may use the time-lapse videos for training purposes and quality control. TLM may offer logistic advantages and flexibility for an IVF laboratory, including remote embryo evaluations and flexible scheduling of procedures and improve laboratory workflow efficiency, allowing embryologists to allocate more time to other essential tasks. However, the impact of TLM on annual treatment capacity and staffing requirements remains uncertain because many costs, e.g. staffing, equipment, and facilities are fixed in IVF laboratories. Therefore, efficiency gains would only translate into lower clinic-level costs if they were large enough to allow for a reduction in staff or a substantial increase in the number of cycles performed per year. Assessing such downstream effects would require a formal budget impact or capacity analysis, which was beyond the scope of the present per-cycle economic evaluation. Given the dynamic nature and rapid evolution of technological innovations in IVF, evaluation strategies that complement conventional RCTs may be warranted. A structured, multidimensional framework could integrate phased introduction of innovations with predefined clinical and economic benchmarks, adaptive trial designs, and the use of multicentre registries to generate evidence in routine practice.

Time measurements for the laboratory procedures were performed at the end of the study period in the IVF laboratory with the largest number of inclusions (Amsterdam UMC) to minimize the influence of learning curves. We acknowledge that conducting time measurements in all five IVF laboratories would have yielded more representative data. Another limitation of our study is that we only report cost-effectiveness for the Geri+ TLM incubator, while more TLM systems are currently available. To some extent, the scenario analyses enable translation of our findings to other culture approaches. We incorporated the findings from a recent RCT reporting a 10-fold reduction in evaluation time to align with modern time-lapse equipment, and modelled extended embryo culture until Day 5. However, our scenario analyses do not account for several clinically relevant differences between Day 3 and Day 5 transfers, including the lower number of embryo transfers required to achieve a pregnancy with a Day 5 strategy (Cornelisse *et al.*, 2024). Furthermore, the most recent Cochrane review suggests that, in good-prognosis patients, blastocyst transfers may improve cumulative LBRs, but are also associated with a higher incidence of premature births (Glujovsky *et al.*, 2026). Given this potential trade-off, a formal cost-effectiveness analysis comparing Day 3 and Day 5 transfer strategies in good and poor prognosis patients is warranted.

Notable strengths of our trial include the use of prospective data from a large RCT on TLM and an economic evaluation based on the 12-month cumulative LBR across all three groups, consistent with the predefined duration of the study. This allows for a meaningful assessment of cost-effectiveness in clinical practice. Extending the follow-up period until all embryos were thawed and transferred would likely have resulted in slightly higher cumulative LBRs. However, as the average number of surplus embryos remaining in storage per patient did not differ between the three groups, we do not expect this to have materially affected the comparative results of the cost-effectiveness analysis. Moreover, restricting the analysis to 12 months preserves internal validity by ensuring that costs and outcomes can be more reliably attributed to the index treatment rather than to subsequent patient-driven treatment decisions.

While considering the potential benefits of TLM, one should acknowledge that, in addition to the substantial initial purchase costs, the use of TLM entails higher ongoing expenses related to the maintenance of TLM incubators and more expensive disposables. The additional burden to society and citizens must be considered in countries which reimburse IVF cycles, since costs for IVF treatments rise as clinics continue to invest in TLM equipment. The ESHRE Capri Workshop Group states that decisions should be based on robust economic analyses (ESHRE Capri Workshop Group, 2015). However, most IVF clinics do not necessarily take national healthcare affordability into account when deciding which technology to use in their laboratory. It should be highlighted that the results of an economic analysis from the perspective of IVF clinics as health care provider may differ from an analysis from a societal perspective. Economic analyses from a societal perspective usually include productivity loss. A limitation of our study is that we did not record productivity loss for couples in the three groups. But it is unlikely that productivity loss would differ substantially between the three strategies, since time to pregnancy within the 1-year follow-up period was comparable and strategies only differed in terms of laboratory procedures (Kieslinger *et al.*, 2023). The number of embryo transfers was also similar across groups, suggesting that costs related to work absence for hospital visits are unlikely to differ. Given the comparable pregnancy outcomes in an otherwise healthy population, societal costs are not expected to vary significantly.

In addition to an economic evaluation from a health care and societal perspective, it is mandatory to also consider a patient perspective when evaluating an innovation. TLM may offer patient-perceived value as couples may benefit from a better understanding of their treatment cycle and increased engagement and emotional attachment through embryo development videos. While meaningful, such non-health benefits are difficult to quantify and were not included in this economic evaluation. The extra costs of add-ons may pose substantial financial burdens on patients seeking infertility treatment. According to a recent study, only 53% of the 88 countries surveyed offer reimbursement for IVF costs (International Federation of Fertility Societies, 2022). Couples do not have endless funds and may have to choose between add-ons or a new IVF cycle. Information from clinic websites regarding the effectiveness of add-ons does not always reliably reflect the current evidence base (van de Wiel *et al.*, 2020). To make informed decisions, couples should be directed to objective sources of information, such as ESHRE ‘The European Society of Human Reproduction and Embryology’ or HFEA ‘The Human Fertility and Embryology Authority’, that provide evidence-based guidance on the effectiveness of add-ons.

## Conclusion

In conclusion, our analysis provides evidence that TLM is unlikely to be cost-effective compared with conventional methods, although it is not clinically inferior and may offer certain advantages. Ensuring the long-term affordability of assisted reproduction for national healthcare systems, healthcare providers, and couples requires careful economic evaluation and consideration of the societal willingness-to-pay threshold for an additional live birth.

## Supplementary Material

hoag034_Supplementary_Data

## Data Availability

Restricted access to the study data can be arranged on request to the corresponding author. Written proposals will be assessed by the SelecTIMO study group. A data sharing agreement including terms and conditions for authorship and publication will have to be signed before the data is made available.
