# Supplementary material for: Cost-effectiveness of time-lapse monitoring with or without the use of embryo selection software compared to routine incubation and selection
Source: Hum Reprod Open. 2026 Apr 22;2026(2):hoag034. doi: 10.1093/hropen/hoag034 (PMC13167187; doi:10.1093/hropen/hoag034)
Supplement: hoag034_Supplementary_Data [file hoag034_supplementary_data.docx]

**Supplementary Figures for ‘Cost effectiveness of time-lapse monitoring with or without the use of embryo selection software compared to routine incubation and selection’**

A


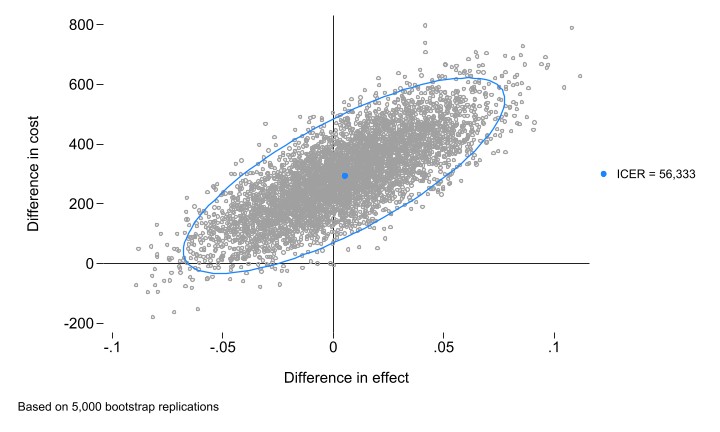


**Supplementary Figure S1: Scenario 1: TLE versus CON**Cost effectiveness plane (A) and cost effectiveness acceptability curve (B)

TLE=time-lapse early embryo viability assessment. CON=control. ICER=incremental cost-effectiveness ratio.


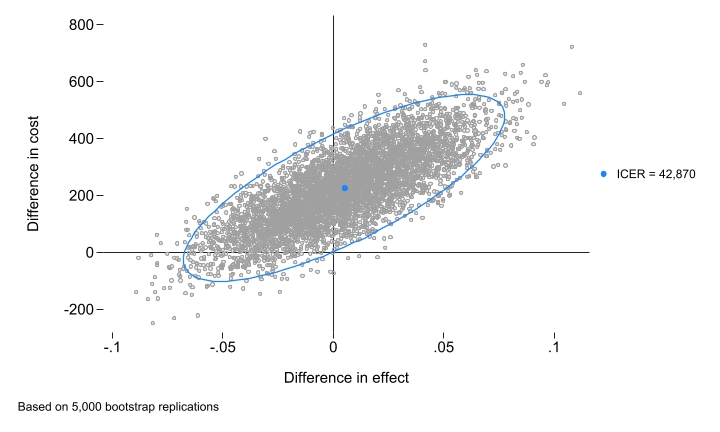


A

**Supplementary Figure S2: Scenario 2: TLE versus CON**Cost effectiveness plane (A) and cost effectiveness acceptability curve (B)

TLE=time-lapse early embryo viability assessment. CON=control. ICER=incremental cost-effectiveness ratio.


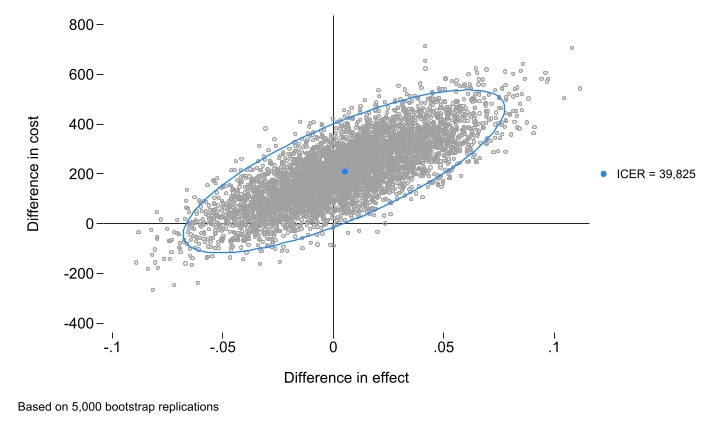


A

**Supplementary Figure S3: Scenario 3: TLE versus CON**Cost effectiveness plane (A) and cost effectiveness acceptability curve (B)

TLE=time-lapse early embryo viability assessment. CON=control. ICER=incremental cost-effectiveness ratio.


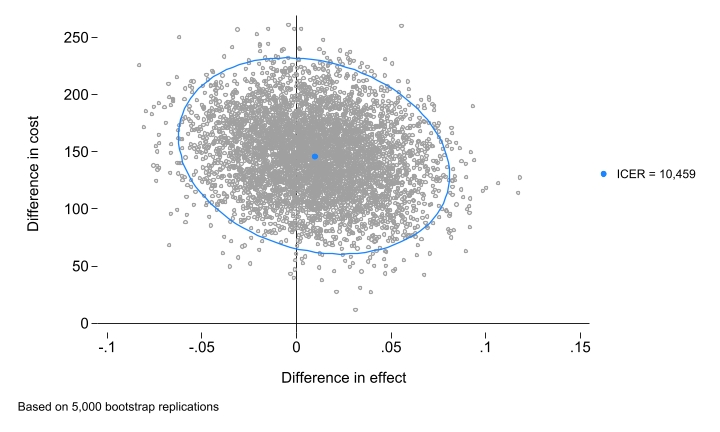


**Supplementary Figure S4: Sensitivity analysis for ongoing pregnancy rate: TLE versus CON**Cost effectiveness plane

No CEAC was prepared as costs over time will always include perinatal and delivery costs.

TLE=time-lapse early embryo viability assessment. CON=control. ICER=incremental cost-effectiveness ratio.


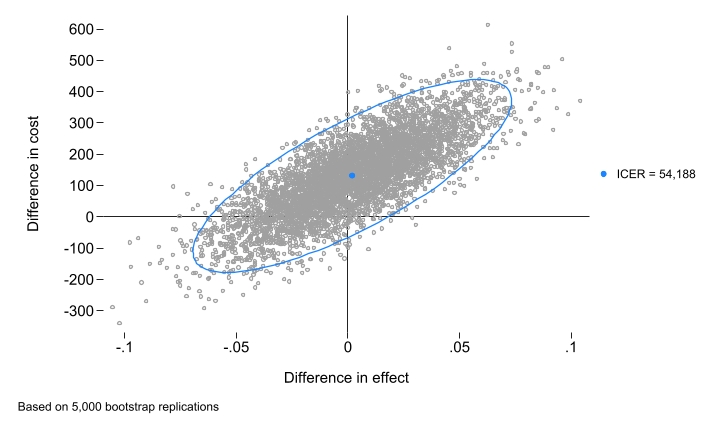


A

**Supplementary Figure S5: Scenario 1: TLR versus CON**Cost effectiveness plane (A) and cost effectiveness acceptability curve (B)

TLR=time-lapse routine. CON=control. ICER=incremental cost-effectiveness ratio.


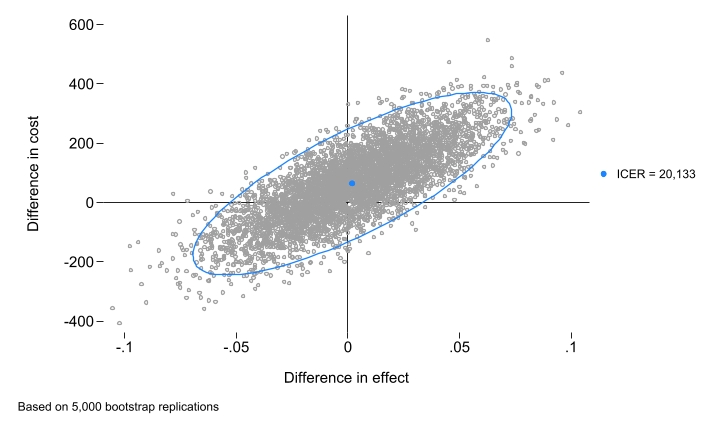


A

**Supplementary Figure S6: Scenario 2: TLR versus CON**Cost effectiveness plane (A) and cost effectiveness acceptability curve (B)

TLR=time-lapse routine. CON=control. ICER=incremental cost-effectiveness ratio.


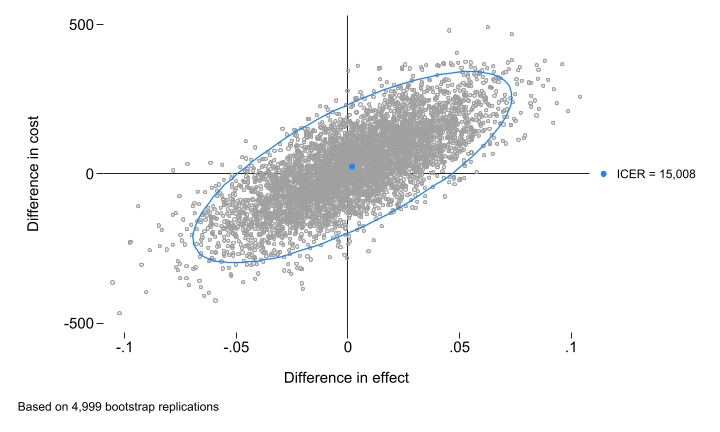


A

**Supplementary Figure S7: Scenario 3: TLR versus CON**Cost effectiveness plane (A) and cost effectiveness acceptability curve (B)

TLR=time-lapse routine. CON=control. ICER=incremental cost-effectiveness ratio.


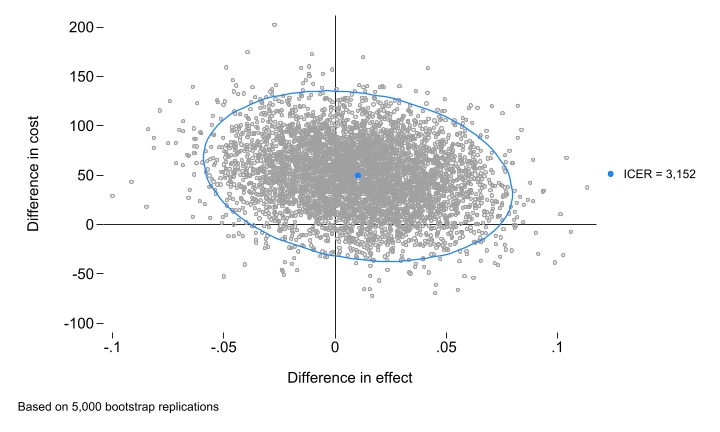


**Supplementary Figure S8: Sensitivity analysis with ongoing pregnancy as outcome: TLR versus CON**Cost effectiveness plane

No CEAC was prepared as costs over time will always include perinatal and delivery costs.

TLR=time-lapse routine. CON=control. ICER=incremental cost-effectiveness ratio.

**Supplementary Table S1: Estimates of total costs in the main model, including estimates for costs per stratification factors laboratory and cycle number**

| \| ------------------------------------------------------------------------------- \| \| \| \| \| \| \|  \| \| --- \| --- \| --- \| --- \| --- \| --- \| --- \| --- \| \| \| TLE versus CON \| \| \| \| \| \|  \|  \| \| \| Estimate [95% conf. interval] \| \| \| \| \| \| \| \| \| --------------+------------------------------------------------------- \| \| \| \| \| \| \| \| \| RANDOMIZATION \| \| \| \|  \|  \|  \|  \|  \| \| 0 \| 2559.570 2387.084 2733.057 \| \| \| \| \| \| \| \| \| 1 \| 2796.764 2594.039 2999.690 \| \| \| \| \| \| \| \| \| \| \| \| \|  \|  \|  \|  \|  \| \| Laboratory \| \| \| \|  \|  \|  \|  \|  \| \| 1 \| 2533.829 2302.662 2764.995 \| \| \| \| \| \| \| \| \| 2 \| 2407.799 1966.340 2849.257 \| \| \| \| \| \| \| \| \| 3 \| 2443.110 2146.235 2739.984 \| \| \| \| \| \| \| \| \| 4 \| 2661.129 2462.568 2859.689 \| \| \| \| \| \| \| \| \| 5 \| 2594.195 2150.523 3037.866 \| \| \| \| \| \| \| \| \| \| \| \| \|  \|  \|  \|  \|  \| \| Cycle \| \| \| \|  \|  \|  \|  \|  \| \| 1 \| 2670.058 2540.640 2799.477 \| \| \| \| \| \| \| \| \| 2 \| 2662.138 2212.366 3111.909 \| \| \| \| \| \| \| \| \| 3 \| 2707.736 1866.863 3548.608 \| \| \| \| \| \| \| \| \| ---------------------------------------------------------------------- \| \| \| \| \| \| \| \| \|  \|  \|  \|  \|  \|  \|  \|  \| \|  \|  \|  \|  \|  \|  \|  \|  \| \| ---------------------------------------------------------------------- \| \| \| \| \| \| \| \| \| \| TLR versus CON \| \| \| \| \| \|  \|  \| \| \| Estimate [95% conf. interval] \| \| \| \| \| \| \| \| \| --------------+------------------------------------------------------- \| \| \| \| \| \| \| \| \| RANDOMIZATION \| \| \| \|  \|  \|  \|  \|  \| \| 0 \| 2560.101 2383.523 2732.68 \| \| \| \| \| \| \| \| \| 1 \| 2615.308 2428.625 2800.991 \| \| \| \| \| \| \| \| \| \| \| \| \|  \|  \|  \|  \|  \| \| lab \| \| \| \|  \|  \|  \|  \|  \| \| 2 \| 2486.492 2236.895 2736.089 \| \| \| \| \| \| \| \| \| 3 \| 2459.608 2051.231 2867.986 \| \| \| \| \| \| \| \| \| 4 \| 2517.911 2290.337 2809.485 \| \| \| \| \| \| \| \| \| 5 \| 2663.429 2488.403 2838.454 \| \| \| \| \| \| \| \| \| 6 \| 2732.044 2246.798 3217.29 \| \| \| \| \| \| \|  \| \| \| \| \| \|  \|  \|  \|  \|  \| \| cycle \| \| \| \|  \|  \|  \|  \|  \| \| 1 \| 2681.793 2552.971 2810.615 \| \| \| \| \| \| \| \| \| 2 \| 2690.301 2251.938 3128.663 \| \| \| \| \| \| \| \| \| 3 \| 2714.605 1196.505 4232.706 \| \| \| \| \| \| \| \| \| ---------------------------------------------------------------------- \| \| \| \| \| \| \| \| \|  \|  \|  \|  \|  \|  \|  \|  \| |
| --- | --- | --- | --- | --- | --- | --- | --- | --- | --- | --- | --- | --- | --- | --- | --- | --- | --- | --- | --- | --- | --- | --- | --- | --- | --- | --- | --- | --- | --- | --- | --- | --- | --- | --- | --- | --- | --- | --- | --- | --- | --- | --- | --- | --- | --- | --- | --- | --- | --- | --- | --- | --- | --- | --- | --- | --- | --- | --- | --- | --- | --- | --- | --- | --- | --- | --- | --- | --- | --- | --- | --- | --- | --- | --- | --- | --- | --- | --- | --- | --- | --- | --- | --- | --- | --- | --- | --- | --- | --- | --- | --- | --- | --- | --- | --- | --- | --- | --- | --- | --- | --- | --- | --- | --- | --- | --- | --- | --- | --- | --- | --- | --- | --- | --- | --- | --- | --- | --- | --- | --- | --- | --- | --- | --- | --- | --- | --- | --- | --- | --- | --- | --- | --- | --- | --- | --- | --- | --- | --- | --- | --- | --- | --- | --- | --- | --- | --- | --- | --- | --- | --- | --- | --- | --- | --- | --- | --- | --- | --- | --- | --- | --- | --- | --- | --- | --- | --- | --- | --- | --- | --- | --- | --- | --- | --- | --- | --- | --- | --- | --- | --- | --- | --- | --- | --- | --- | --- | --- | --- | --- | --- | --- | --- | --- | --- | --- | --- | --- | --- | --- | --- | --- | --- | --- | --- | --- | --- | --- | --- | --- | --- | --- | --- | --- | --- | --- | --- | --- | --- | --- | --- | --- | --- | --- | --- | --- | --- | --- | --- | --- | --- | --- | --- | --- | --- | --- | --- | --- | --- | --- | --- | --- | --- | --- | --- | --- | --- | --- | --- | --- | --- | --- | --- | --- | --- | --- | --- | --- | --- | --- | --- | --- | --- | --- | --- | --- | --- | --- | --- | --- | --- | --- | --- | --- | --- | --- | --- | --- | --- | --- | --- | --- | --- | --- | --- | --- | --- | --- | --- | --- | --- | --- | --- | --- | --- | --- | --- | --- | --- | --- | --- | --- | --- | --- | --- | --- | --- | --- | --- | --- | --- | --- | --- | --- | --- | --- | --- | --- | --- | --- | --- | --- | --- | --- | --- | --- | --- | --- | --- | --- | --- | --- | --- | --- | --- | --- | --- | --- | --- | --- | --- | --- | --- | --- |

TLE, time-lapse early embryo viability assessment; TLR, time-lapse routine; CON, control.
